# Secondary Metabolites Coordinately Protect Grapes from Excessive Light and Sunburn Damage during Development

**DOI:** 10.3390/biom12010042

**Published:** 2021-12-28

**Authors:** Joanna M. Gambetta, Valentina Romat, Leigh M. Schmidtke, Bruno P. Holzapfel

**Affiliations:** 1 School of Agricultural, Environmental and Veterinary Sciences, National Wine and Grape Industry Centre, Charles Sturt University, Locked Bag 588, Wagga Wagga, NSW 2678, Australia; romatvalentina@gmail.com (V.R.); lschmidtke@csu.edu.au (L.M.S.); 2 Waite Research Institute, School of Agriculture, Food and Wine, University of Adelaide, PMB1, Glen Osmond, SA 5064, Australia; 3 Wagga Wagga Agriculture Institute, New South Wales Department of Primary Industries, Wagga Wagga, NSW 2650, Australia; Bruno.Holzapfel@dpi.nsw.gov.au

**Keywords:** acclimation, carotenoids, defoliation, excess sunlight, polyphenols, sunburn, *Vitis vinifera*, volatile compounds

## Abstract

Sunburn is a physiological disorder that reduces grape quality and vineyard yield. It is the result of excessive sunlight and high temperatures. As climate change continues to increase air temperatures, reports of sunburn damage in vineyards worldwide are becoming more frequent. Grapes produce secondary metabolites (carotenoids, polyphenols and aroma compounds) to counter photooxidative stress and acclimate to higher radiation environments. This study evaluated changes in these compounds in during ripening when grapes were exposed post-flowering (ED) and at véraison (LD), and compared them to a nondefoliated control (ND). ND contained more α-terpineol and violaxanthin, and the defoliated treatments contained more zeaxanthin, β-carotene, C_6_ compounds and flavonoids. ED berries adapted better to higher-light environments, displayed larger changes in secondary metabolite concentrations and lower levels of sunburn damage than LD berries did. The composition of berries with increasing sunburn damage was evaluated for the first time. Berries with no damage had the lowest concentrations of flavonoids and oxidized glutathione, and the highest concentrations of chlorophyll and α-terpineol. As damage increased, destruction of photosynthetic pigments, increase in polyphenols and loss of aroma compounds were evidenced. A significant effect of temperature and developmental stage on grape composition was also observed. This study provides a holistic overview of changes in secondary metabolites experienced by grape berries when exposed to excessive light, how these vary along development and how they affect sunburn incidence.

## 1. Introduction

Canopy management practices such as defoliation are commonly performed in cool viticultural regions to increase air flow, improve spray penetration, decrease disease pressure, reduce vine yield and improve fruit composition [1]. However, by opening the bunch area, radiative exposure at the grape level is increased, and the sudden exposure of shaded fruit (without prior acclimation of the tissue) might lead to photooxidative damage and sunburn, especially when ambient temperature is excessive [2]. Sunburn damage adversely affects grape (and wine) composition, leading to significant economic losses of up to 50% crop value and upwards of 60% bottle price due to yield and quality reduction [2].

Although solar radiation is critical to the development of plants and fruits, the loss of shade can result in excessive sunlight and the production of reactive oxygen species (ROS) and hydrogen peroxide that can damage the fruit’s photosystems and cause photooxidation and membrane lipid peroxidation [2,3,4]. Fruits like grape berries and apples have evolved enzymatic and nonenzymatic adaptation strategies to acclimate and attenuate the effects of excessive radiation, which include the glutathione–ascorbate cycle, non-photochemical quenching (NPQ) and the accumulation of secondary metabolites [2].

Secondary metabolites such as the carotenoids and polyphenols are well-established sunscreen compounds that are used to protect the berry’s photosynthetic apparatus from photo- and thermal oxidation and acclimate to changing levels of sunlight [5,6]. They function as antioxidants capable of scavenging ROS and quenching singlet oxygen (^1^O_2_) and triplet chlorophyll (^3^Chl*), and screening light at different wavelengths. For instance, the xanthophyll cycle, whereby violaxanthin, anteraxanthin and zeaxanthin are interconverted in the presence of ascorbate and a pH gradient, constitutes one of the main forms of NPQ. It is quickly activated in response to light stress, and dissipates excess light energy as heat [7,8]. The presence of conjugated double-bond systems in the polyphenol structure favours the absorption of low-energetic radiation; for example, the flavonols absorb light at 280 nm, which coincides with UV radiation [9]. Furthermore, polyphenol synthesis is strongly induced by UV radiation, which activates a series of enzymes in the phenylpropanoid pathway, and several studies have confirmed a marked increase in the concentration of these compounds in sun-exposed grapes. More recently, aroma compounds (e.g., terpenes, alcohols and aldehydes) have also been implicated in the protection of grapes against photooxidation, but the mechanism of action is not clear yet [10,11,12]. Studies on the effect of light and UV on berry composition have demonstrated that their concentration, like that of carotenoids and polyphenols, is upregulated by higher radiation levels, and that they could also be functioning as antioxidants and sunscreens [8,13].

While most studies have concentrated on specific groups of compounds, changes in the light environment cause an extensive rearrangement of the whole transcriptome [14]. This points to a coordinated effort to protect the berry and acclimate to excessive radiation by utilizing different groups of secondary metabolites simultaneously that seem to be strongly linked to berry development [1,5,14]. Different genes are active at different developmental stages, and fruits display a changing susceptibility to excess sunlight as they develop. Young fruits are still photosynthetically active, and their responses to light are more akin to those of leaves than to that of mature fruit. As berries ripen and chloroplasts are dismantled, the capacity to produce carotenoids is progressively lost and acclimation strategies change from a chloroplast-based defence to the accumulation of phenolics and antioxidants [13,15]. Although there is an extensive literature on the response of individual compounds such as the polyphenols to an altered light environment, there is little information on how the responses of all these important metabolites (polyphenols, carotenoids and aroma compounds) are integrated together as well as with the temporal metabolic changes that are typical of fruit development.

If the berry’s antioxidant systems fail, accumulation of ROS ultimately leads to the destruction of the photosynthetic apparatus and sunburn [16]. Sunburn is a physiological disorder that impacts the composition and visual aspect of the fruit and leads to significant losses of quality and yield in a range of crops including grapes and apples. Depending on the severity of the damage, sunburn can be catalogued as either sunburn browning (SB), which manifests as brown lesions on the berry surface, or as sunburn necrosis (SN), which manifests as cracked and desiccated fruit [6]. Sunburn results from a combination of high light intensity, ultraviolet radiation and excessive heat, which is modulated by a series of factors including environmental conditions, grape variety, fruit developmental stage, water stress and canopy management [17,18].

Climate change, compounded with a higher incidence of heatwaves in cool viticultural areas such as Orange (Australia) and Champagne, has significantly increased the frequency at which sunburn is observed in these regions [2]. Since fungal rots are still an important problem in these areas, defoliation remains one of the best management strategies to decrease disease pressure [19]. However, a better understanding of the underlying principles and optimal timing of this practice is necessary to minimize sunburn and crop loss. There is little information on the changes in composition, particularly in regards to aroma compounds, related to adaptation to higher-light environments at different developmental stages and varying levels of sunburn. Therefore, the aims of this study were to (i) evaluate and compare the composition of Chardonnay berries defoliated after flowering with that of berries defoliated at véraison and not defoliated in two vineyards located at different altitudes; (ii) evaluate how these compositional changes and adaptation mechanisms evolve during ripening; (iii) at harvest, rate the incidence of sunburn in these samples and evaluate the compositional changes derived from increasing degrees of sunburn browning.

## 2. Materials and Methods

### 2.1. Experimental Design

Field trials were conducted over the 2018–2019 growing season in two commercial vineyards: Cumulus (CUM, 32°58′47.4″ S, 148°57′42.8″ E) and Balmoral (BAL, 33°16′03.9″ S, 148°59′59.0″ E), located at two different elevations (612 m.a.s.l. and 884 m.a.s.l., respectively) in the Orange wine region, New South Wales, Australia. The trial used Chardonnay grapevines (clone 110V1) planted in 2000 on own roots (CUM) and grafted onto Shiraz in 1995 (BAL). Row orientation was North–South, with a 3.0 m spacing between rows and 2.0 m in-row spacing at both sites. The vines were trellised to a single bilateral cordon with a vertical shoot-positioning system. Both sites featured red brown earth soil types [20]. Vines were drip irrigated in 3-h intervals as needed.

Treatments were comprised of a control (no defoliation, ND), an early defoliation treatment (ED, E-L27 [21]) and a late defoliation treatment (LD, E-L35). Early defoliation was performed on 6th December 2018, at both vineyards (17 days after flowering, DAF, at CUM and 9 DAF at BAL), and late defoliation on 8th January 2019 in CUM (50 DAF) and January 23rd in BAL (56 DAF). Consistent with local practice, all leaves and lateral shoots were manually removed in the fruiting zone (45 cm above the cordon) on the east side of the canopy. The fruiting zone of ED and LD was kept exposed for the duration of the trial through continuous leaf removal. Each treatment consisted of three replicates laid out in a completely randomized arrangement across three rows, separated by two buffer rows (which were also defoliated). Each replicate included six vines, with sampling constrained to the four central vines, leaving the two external vines as buffers. All treatments were harvested at commercial maturity (~21 °Brix) on 4th February 2019 in CUM, and on 1st March 2019 in BAL. Key vineyard information and trial dates are summarized in Supplementary Table S1.

### 2.2. Climatic Monitoring and Vineyard Measurements

Canopy mesoclimate (temperature and relative humidity) was assessed every 10 min with a dual-channel logger (TinyTag TGP-4500; Hastings Data Loggers, Port Macquarie, NSW, Australia) located in the middle of the canopy. Ultraviolet radiation and photosynthetically active radiation (PAR) were measured using a UV sensor (SU-100-SS, Apogee, Campbell Scientific Australia Pty Ltd., Garbutt, QLD, Australia) and a sun quantum sensor (Apogee, Campbell Scientific Australia Pty Ltd., Garbutt, QLD, Australia). UV and quantum sensor signals were scanned and recorded every 30 min. Thermocouples [22] connected to two data acquisition systems (AM-25T and CR-1000, Campbell Scientific, Logan, UT, USA) recorded the temperature of eight bunches per treatment (24 in total) every 10 min. Sunlight interception at the bunch level was measured using a light-sensitive tape (Optoleaf Y-1W, Taisei Fine Chemical Co., Ltd., Tokyo, Japan) with calibration curves provided by the supplier. All sensors and tapes were located only in the middle row of the experimental area in each vineyard. Other environmental variables were calculated using data from local weather stations. Additional details related to other measurements performed in the vineyard, such as leaf area (LA) and midday stem water potential (SWP), appear in Supplementary Information.

### 2.3. Sample Collection

Grape samples (n = 170) were collected every two weeks from E-L29 onwards only from the exposed facet of bunches located on the eastern side of the canopy. Fifty berries (transported on ice) were used to determine basic chemical parameters. The remainder of the sample was immediately flash frozen in the field using liquid nitrogen and was used for carotenoid, total polyphenol and volatile compound analysis. At harvest, fifteen bunches per treatment replicate were randomly chosen from the eastern side of the canopy and reserved for sunburn assessment.

### 2.4. Sunburn Damage Severity

Grapes were manually destemmed, grapes per bunch were counted and then segregated according to damage type and level of damage. Sunburn damage was categorized as no damage (SB0), sunburn browning (SB) or sunburn necrosis (SN). Sunburn browning was further qualified with relation to intensity as SB1 (tan berries), SB2 (brown berries) and SB3 (dark brown berries), with SB3 berries exhibiting the highest level of damage. The number of berries in each category per bunch was recorded and expressed as % damaged berries. Grapes from each SB damage class (SB0–SB3) were aggregated and analysed for free volatiles, polyphenols and carotenoids as described below.

### 2.5. Chemical Analyses

Reagents: All solvents were of liquid chromatography (LC) purity unless otherwise stated. Acetonitrile, methanol, chloroform and ethyl acetate were purchased from Merck (Kilsyth, VIC, Australia). Reference compounds for free volatile compound analysis (purity ≥97%), 2-octanol, trans-β-Apo-8′-carotenal (purity ≥96%), chlorophyll *a* (Chl *a*, ≥95%), β-carotene (≥95%), formic acid (American Chemical Society reagent, ≥98%), trifluoroacetic acid (Reagent Plus^®^, 99%), L-ascorbic acid (reagent grade, ≥98%), quercetin-3-*O*-glucoside (analytical standard, 98%), corticosterone (≥98%), ampicillin trihydrate (analytical standard, ≥98%), L-tyrosine (≥99%), L-phenylalanine (≥99%), L-glutathione reduced (BioReagent, ≥98.0%) and triethylamine (TEA, ≥99%) were purchased from Sigma-Aldrich (Castle Hill, NSW, Australia). Additional details regarding materials and preparation of solutions appear in Supplementary Information.

Basic grape chemistry: Total soluble solids (TSS), titratable acidity (TA), pH and yeast assimilable nitrogen (YAN) were measured as previously described [23,24]. The ammonium and α-amino acid concentrations of the juice were determined using a commercially available enzymatic assay kit, designed for an Arena discrete analyser (Thermofisher, Scoresby, VIC, Australia). YAN concentration was then calculated from the ammonium and free amino N [24]. Further details on these analyses and equipment can be found in Supplementary Information.

Polyphenol determination by liquid chromatography with triple-quadrupole mass spectrometry (LC-QqQ-MS): Polyphenol content was determined using the skin of twenty berries, previously freeze-dried and ground, according to Gouot et al. [22].

Carotenoid and chlorophyll extraction and ultra-performance liquid chromatography method with diode array detection (UPLC-DAD) analysis: Carotenoid and chlorophyll analysis was carried out as outlined previously by Wehrens et al. [25]. Additional details regarding sample extraction and preparation appear in Supplementary Information. Extracts and standards were analysed by UPLC-DAD as described by Lashbrooke et al. [26].

Analysis of free volatile compounds by headspace solid-phase microextraction and gas chromatography mass spectrometry (HS-SPME-GCMS): Two grams of previously deseeded and ground berry powder were weighed into a 10 mL SPME vial (Agilent, Palo Alto, CA, USA). Two millilitres of a phosphate–citrate buffer (pH 5), 40 μL of an ascorbic acid solution (50 g/L), 20 μL of internal standard (2-octanol (5.08 mg/L), d3-linalool (1.89 mg/L), d13-1-hexanol (5.0 mg/L) and d12-hexanal (50.1 mg/L) in methanol) and 1 g NaCl were added to each vial, briefly vortexed and capped. Samples were analysed by HS-SPME-GCMS as previously described [23].

### 2.6. Statistical Analyses

All statistical analyses were carried out using R v4.0.1 (Rstudio Team, 2020) and Matlab v2020a (The Mathworks, Natick, MA, USA). One- and two-way ANOVA (significance level of 5%) were used to explore the differences in climatic data, vineyard parameters, damage levels, basic chemistry and sunburn damage between vineyards and treatments. The effects of the treatment timing (factor XA), developmental stage (DAF; factor XB) and their interaction (factor XAB) on berry composition were investigated using analysis of variance–simultaneous component analysis (ASCA) [27]. Data blocks consisted of polyphenols (PP), carotenoids (CAR) and free volatiles (FV). Log2 fold change was calculated as follows: log2(mean treatment/mean control) for each sampling point and vineyard. Differentially expressed metabolites according to SB damage were clustered using the k-means algorithm, Euclidian-based distances and 100 iterations to determine overall cluster consistency. Cluster number (K = 5) were preliminarily determined using hierarchical cluster analysis with a distance of 0.5. Individual plots of each metabolite were examined according to SB damage and evaluated using silhouette plots. Matlab scripts used for ASCA can be found at https://www.chem.uniroma1.it/romechemometrics/research/algorithms/ (accessed on 26 October 2021), and information on data pre-treatment prior to ASCA can be found in Supplementary Information.

## 3. Results

### 3.1. Effect of Different Light Exposure Timing on Composition

There were no significant differences between bunch number, bunch weight, yield or most basic chemistry parameters (TSS, TA, pH and YAN) between treatments at either vineyard at harvest (Supplementary Table S2). Significant differences were observed only between vineyards: berry weight, TA and TSS were higher at BAL while pH and YAN were higher at CUM. Bunch number was significantly higher at CUM than at BAL, but bunches at CUM were significantly smaller (CUM, 90 g; BAL, 124g). With regards to LA, canopies were slightly larger at BAL (11.1 m^2^ ± 3.3, ND) than at CUM (8.0 m^2^ ± 0.6, ND). LA/kg ratios were significantly different only between ND and LD treatments and only at BAL.

**Figure 1:**
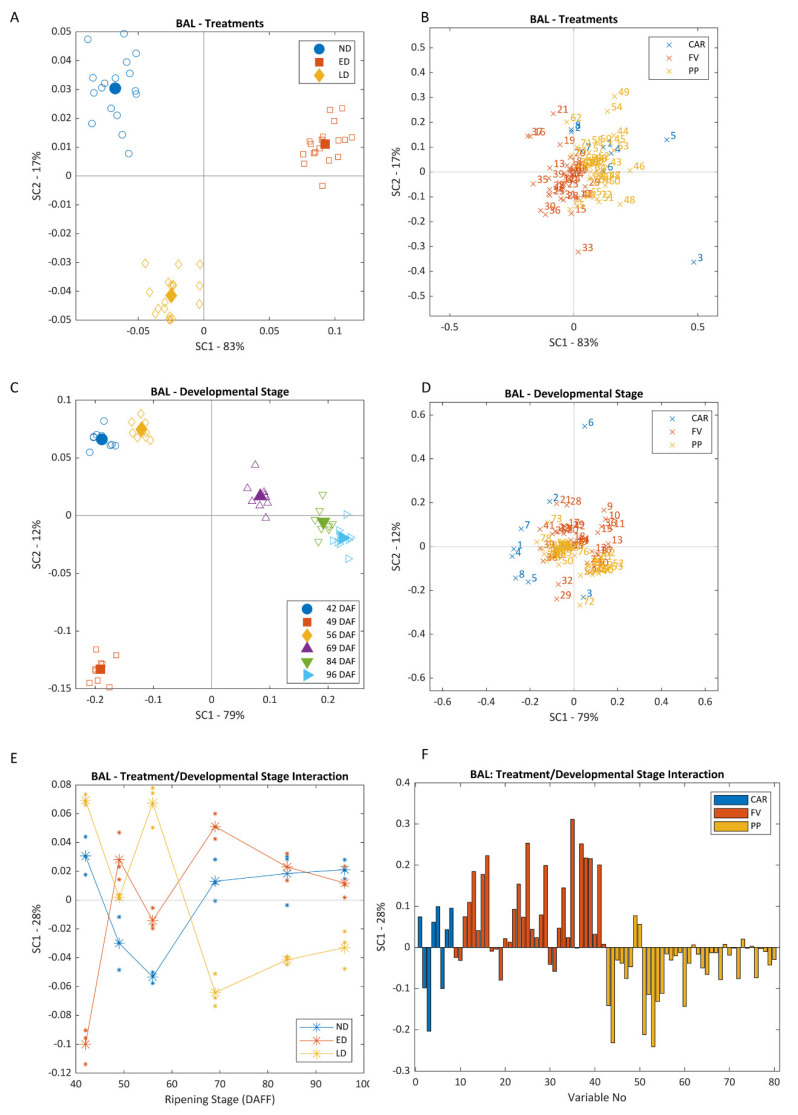
Scores (**A**,**C**) and loadings (**B**,**D**) ASCA plots for the first and second SC, and longitudinal scores (**E**) and loadings (**F**) plots for SC1, for BAL vineyard. Plots (**A**,**B**) correspond to factor Treatments, (**C**,**D**) to factor Developmental Stage, and (**E**,**F**) to the model’s interaction term. A list of variables used in loadings plots (**B**,**D**,**F**) can be found in Supplementary Table S5. SC stands for simultaneous component; ND, no defoliation; ED, early defoliation; LD, late defoliation; CAR, carotenoids; FV, free volatiles; PP, polyphenols; DAF, days after flowering. Filled symbols indicate group centroids (**A**,**C**).

**Figure 2:**
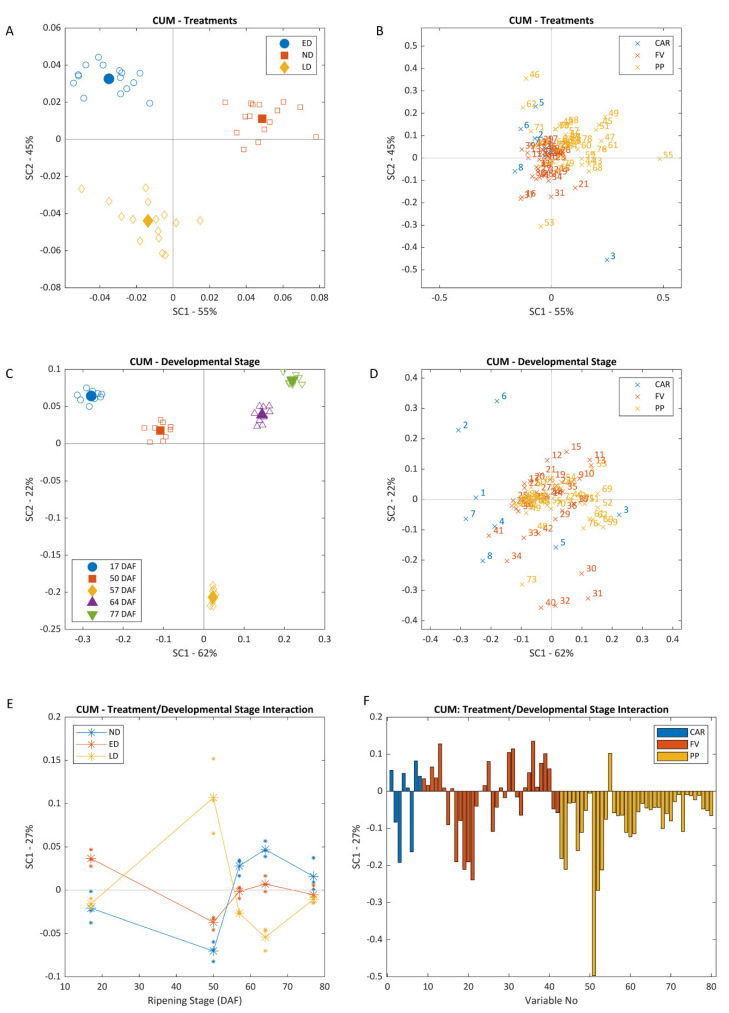
Scores (**A**,**C**) and loadings (**B**,**D**) ASCA plots for the first and second SC, and longitudinal scores (**E**) and loadings (**F**) plots for SC1, for CUM vineyard. Plots (**A**,**B**) correspond to factor Treatments, (**C**,**D**) to factor Developmental Stage and (**E**,**F**) to the model’s interaction term. List of variables used in loadings plots (**B**,**D**,**F**) can be found in Supplementary Table S5. SC stands for simultaneous component; ND, no defoliation; ED, early defoliation; LD, late defoliation; CAR, carotenoids; FV, free volatiles; PP, polyphenols; DAF, days after flowering. Filled symbols indicate group centroids (**A**,**C**).

ANOVA–simultaneous component analysis (ASCA) and permutation testing determined that all three experimental factors (XA, exposure timing; XB, developmental stage and XAB, interaction timing*developmental stage) were significant at a *p*-value of 0.05 for both sites (Figure 1 and Figure 2). Factors XA, XB and XAB accounted each for 10.2%, 71.3% and 13.5% of the total data variance at BAL, and 3.6%, 80.1%, and 10.4% at CUM. Even though most of the variation in the data was explained by factor XB, samples could still be discriminated according to XA, with the same pattern repeated at both vineyards. As berries were exposed at different times during their development, by harvest, there were significant differences (*p* < 0.0001) between the total amount of light intercepted by bunches in each treatment across both vineyards. Average cumulative radiation at the bunch level was of 332 ± 49 (no exposure, ND), 684 ± 267 (early exposure, ED), 427 ± 132 (late exposure, LD) at BAL and 320 ± 102 (ND), 973 ± 208 (ED), and 758 ± 199 mol/m^2^ (LD) at CUM. Differences in the amount of light accumulated affected in turn the composition of berries in each treatment, which could be discriminated according to their concentration of the different secondary metabolites examined.

As can be observed in both Scores plots (Figure 1A and Figure 2A), ND samples were located in the top left corner, while ED samples clustered to the right side of the plot and LD samples were located in the lower left corner of the plot. The main variables driving this separation along the positive side of SC1 were β-carotene, laricitrin, myricetin (M)-*O*-gal and -gln, kaempferol (K)-3-*O*-gal and quercetin (Q)-3-*O*-gal, which were higher in ED samples. 1-Heptanol, 1-octanol and benzaldehyde, which were higher in ND and LD samples, were responsible for separations along the negative side of SC1 (Figure 1B and Figure 2B). Defoliated samples (ED and LD) were separated from ND due to lower levels of α-terpineol, naringenin chalcone, K-3-*O*-gln, violaxanthin and pheophytin (positive side of SC2), and higher levels of zeaxanthin, *E*-3-hexenol, 2-ethyl-1-hexanol, furfural, 1-hexanol and *Z*-resveratrol (negative side of SC2).

**Figure 3:**
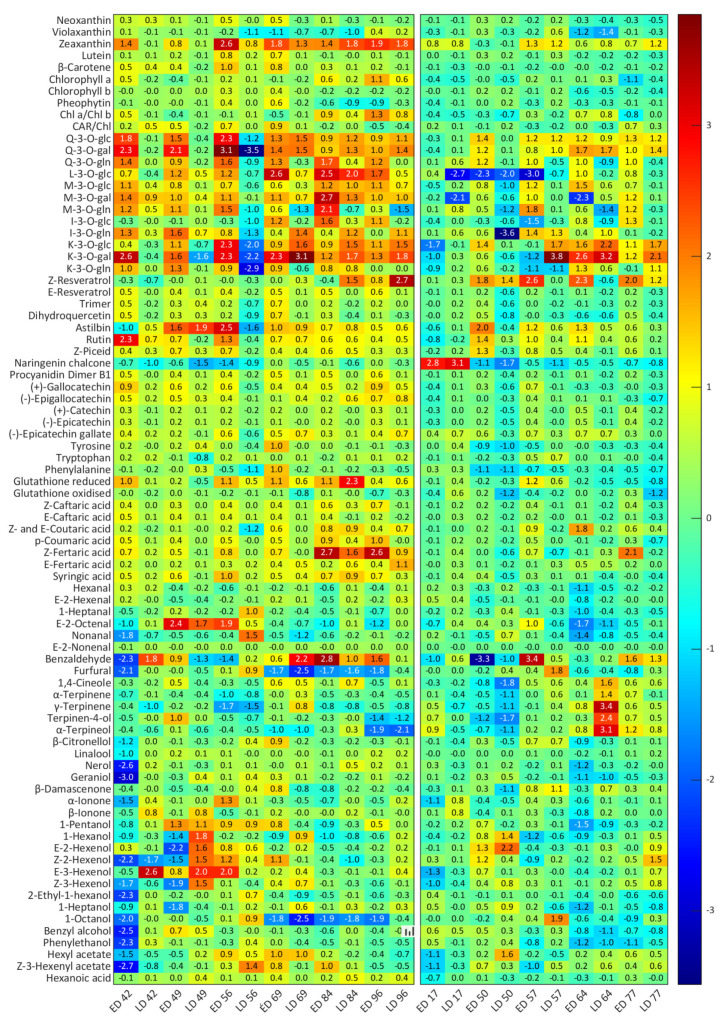
Impact of defoliation timing on carotenoids, polyphenols and free volatile compounds during development at both vineyards. Heatmaps represent log2 fold change (ED/ND and LD/ND) at each developmental stage. Blue and red colours indicate lower and higher metabolite concentration in ED and LD samples, respectively. BAL (left) and CUM (right). ND, no defoliation; ED, early defoliation; LD, late defoliation; Q, quercetin; L, laricitrin; I, isorhamnetin; K, kaempferol; M, myricetin; Glu, glucoside; Gal, galactoside; Gln, gluconoride. Early and late defoliation treatments were conducted at BAL at 9 and 56 DAF, respectively, and at 17 and 50 DAF at CUM.

A clear separation of samples with respect to developmental stage (XB) was evident at both vineyards when plotting SC1 and SC2 scores (Figure 1C and Figure 2C). At BAL, SC1 separated pre- (left) from post-véraison samples (right), with this separation being mainly driven by neoxanthin, lutein, β-carotene, Chl *b*, pheophytin, *Z*-3-hexenyl acetate and phenylethanol, all higher in pre-véraison samples; and 1-heptanal, nonanal, K-3-*O*-glc and -gal, which were higher in post-véraison samples. Separation along the SC2-axis was mainly due to Chl *a*, violaxanthin, α-terpineol, β-ionone and hexanal on the positive side, and 1-pentanol, zeaxanthin, *Z*-2-hexenol and β-carotene on the negative side (Figure 2D and Figure 3D). At CUM, a similar pattern was observed, although separation of pre-véraison samples was also driven by higher contents of violaxanthin, Chl *a* and *Z*-3-hexenyl acetate, lutein and neoxanthin, whilst riper samples contained more zeaxanthin, astilbin, rutin, K-3-*O*-glc and tyrosine. In general, the concentration of all aldehydes increased with ripening regardless of exposure timing, although concentrations of *E*-2-nonenal, 1-heptanal, hexanal and *E*-2-hexenal were significantly lower in ED samples than in ND and LD samples at BAL by harvest. At both vineyards, samples collected on 15 January (49 DAF, 5.2 °Brix, BAL; 57 DAF, 14 °Brix, CUM) were distinctly separated from all others along SC1. Compounds driving this separation varied according to the samples’ maturity levels—at BAL this separation was mainly driven by Chl *b*, and at CUM by Chl *b*, violaxanthin, 1-hexanol, hexyl acetate, *E*-2-, *E*-3- and *Z*-2-hexenol, and oxidized glutathione.

The evolution of sample composition according to ripening stage and treatment is shown in the longitudinal plots of XAB SC1 (Figure 1E and Figure 2E). According to these plots, ND had an intermediate profile when compared to ED and LD, but its behaviour was more akin to that of ED. At both vineyards, ED and LD appear at opposite sides of the SC1-axis, with an inflexion point at véraison that inverts their profiles. These trends persisted in BAL throughout ripening, although at CUM, all groups of samples were clustered together, close to the origin at harvest. At both vineyards, SC1 was correlated on the positive side to nonanal and phenylethanol, and on the negative side to zeaxanthin, K, Q-3-*O*-gal, isorhamnetin-3-*O*-gln and rutin (Figure 1F and Figure 2F). At BAL, SC1 was positively correlated to 1-heptanol, 1-octanol, 1-pentanol, benzyl alcohol, geraniol, linalool, benzaldehyde, furfural, and Z-3-hexenyl acetate. SC1 at CUM was also driven on the positive side by 2-ethyl-1-hexanol and *E*-2-hexenol, and on the negative side by α-terpineol, α-terpinene, terpinene-4-ol and 1,4-cineole.

Log2 fold change was used to compare the effect of exposure timing with the non-exposed controls at each sampling point throughout development. Figure 3 shows that zeaxanthin increased by up to 2.6 (BAL) and 1.3 (CUM) log2 fold in ED treatments at both sites, and by up to 1.9 (BAL) and 1.2 (CUM) log2 fold in LD. β-Carotene and lutein increased in ED samples at BAL with respect to the control early on, but these differences disappeared towards harvest. Chl *a* was also responsive to the treatments but had different behaviours at both vineyards—Chl *a* concentration increased in ED samples at BAL whilst it decreased at CUM. As a consequence of defoliation, the concentration of most flavonoids increased several folds in ED samples (up to 3.1 log2 fold), with Q-3-*O*-gal being affected the most. Q-3-*O*-glc, -gal and -gln reacted quickly and similarly regardless of treatment timing, while M-3-*O*-glc, -gal, -gln, and isorhamnetin-3-*O*-glc displayed higher induction levels only in ED, and K-3-*O*-gal and -gln were more responsive in LD. K-3-*O*-gal exhibited the highest rise in concentration of all analysed flavonoids, it was 3.1 log2 fold higher than ND at BAL and 3.8 log2 fold higher at CUM after defoliation. The concentration of all analysed PP decreased in LD samples immediately after exposure, but later recovered. A moderate increase in flavan-3-ols, particularly epigallocatechin and catechin, hydroxycinnamic acids and stilbenes was observed in ED at BAL, although most of these differences disappeared by harvest. A significant increase in *Z*-resveratrol concentration was observed at CUM (2.6 log2, ED) and BAL (2.7 log2, LD). LD also affected furfural (a 0.9 (BAL) and 1.8 (CUM) log2 fold increase with respect to ND was observed immediately after treatment), and 1-octanol concentration (0.9 and 1.9 log2 fold increase at BAL and CUM, respectively).

### 3.2. Effect of Extreme Temperatures on Secondary Metabolite Production

**Figure 4:**
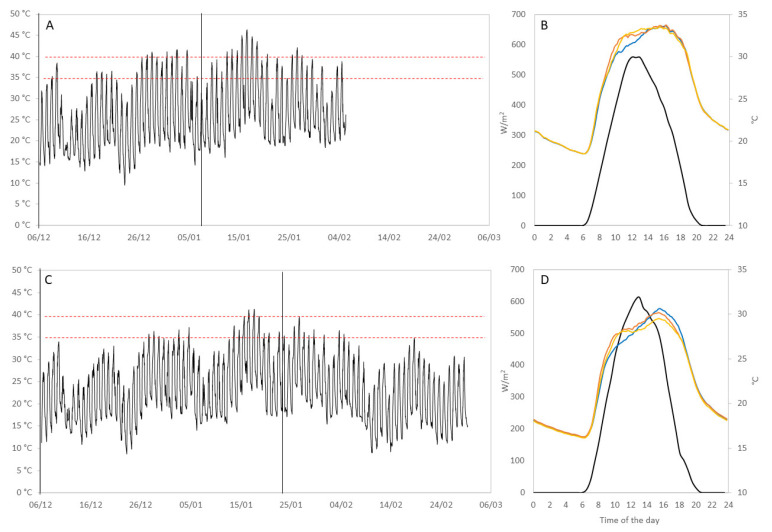
Growing season canopy temperature (**A**,**C**) and average daily solar energy and bunch temperature (**B**,**D**) at CUM (above) and BAL (below). (**A**,**C**) Red broken lines indicate temperatures above 35 and 40 °C, and black vertical lines indicate defoliation treatments (ED, 6/12 at CUM and BAL; LD, 08/01 at CUM and 23/01 at BAL). Canopy temperature was collected between flowering and harvest. (**B**,**D**) Canopy light interception is represented by black lines, and bunch temperatures by blue (ND), orange (ED), and yellow (LD) lines.

Despite being located in the same viticultural region, there were significant differences between the temperature regimes at both vineyards, mainly due to differences in altitude. Average daily canopy temperatures during the trial were of 22.9 (BAL) and 26.2 °C (CUM) (Supplementary Table S3). Average maximum bunch temperatures for BAL were 38.32 (ND), 39.16 (ED) and 37.98 °C (LD) and for CUM were 40.56 (ND), 41.57 (ED) and 40.13 °C (LD) over the developmental period (Figure 4). Maximum temperatures of 47 (ED, 16 January) and 44 °C (LD, 23 January) were at BAL, and of 50 (ED, 16 January) and 51 °C (LD, 27 January) were recorded at CUM. By the end of the season, LD and ND bunches had accumulated significantly less degree days than had ED bunches at BAL. At CUM, ED and LD accumulated significantly more degree days than ND did Paired-sample t-test analysis of ND samples at both vineyards was used to compare the single effect of temperature on berry composition at véraison and harvest (Table 1). Higher temperatures at CUM led to higher contents of zeaxanthin, β-carotene, and Chl a at véraison and harvest, and lower levels of Chl b at harvest. Most flavonoids and hydroxycinnamic acids, and reduced glutathione were higher at CUM than at BAL by harvest; oxidized glutathione, Z-resveratrol, catechin and epicatechin were higher at BAL. Significant differences were observed in the concentrations of several FV compounds between both vineyards at véraison and harvest: CUM had higher contents of the aldehydes hexanal, E-2-hexenal, 1-heptanal, nonanal and furfural, and lower contents of the alcohols 1-hexanol, Z- and E-2-hexenol, Z-3-hexenol, 1-octanol, benzyl alcohol and phenylethanol. Véraison and harvest CUM samples also contained less β-ionone, β-damascenone, β-citronellol, linalool, nerol and geraniol, but higher levels of 1,4-cineole. CUM samples had higher concentrations of γ-terpinene, terpinene-4-ol and α-terpineol than those of BAL samples, but only at véraison.

### 3.3. Level of Sunburn Damage

**Figure 5:**
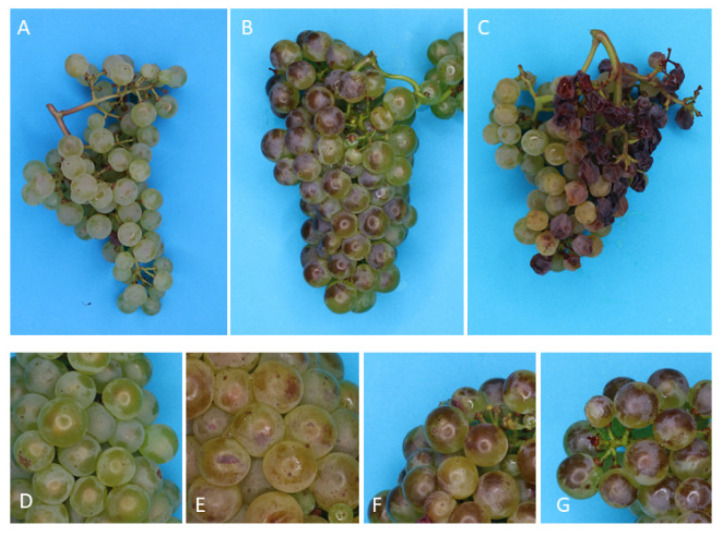
Bunches exhibiting varying degrees of sunburn damage. Above: Bunches exhibiting no damage (**A**), sunburn browning (**B**) and sunburn necrosis (**C**). Below: varying degrees of sunburn browning: (**D**) SB0, (**E**) SB1, (**F**) SB2 and (**G**) SB3.

Four levels of sunburn browning damage (SB0-3) could be distinguished from compositional analysis (see next section), however SB damage was ultimately classified as SB1 and SB2, due to difficulties in accurately differentiating SB2 and SB3 when conducting large amounts of visual assessments. Sunburn damage levels at harvest were significantly different between both vineyards (Table 2)—whilst most of the damage observed at BAL was classified as SB, damage at CUM was mostly classified as SN (Figure 5). At BAL, ED had more SB1 (28%) berries than did ND (10%) and LD (19%), but LD had significantly more berries with a higher level of SB damage (SB2, 26%) than did the other two treatments (2%, ND; 9%, ED). LD (CUM) had a significantly higher proportion of SN berries (22%) than that of both ND and ED.

### 3.4. Composition of SB Levels

**Figure 6:**
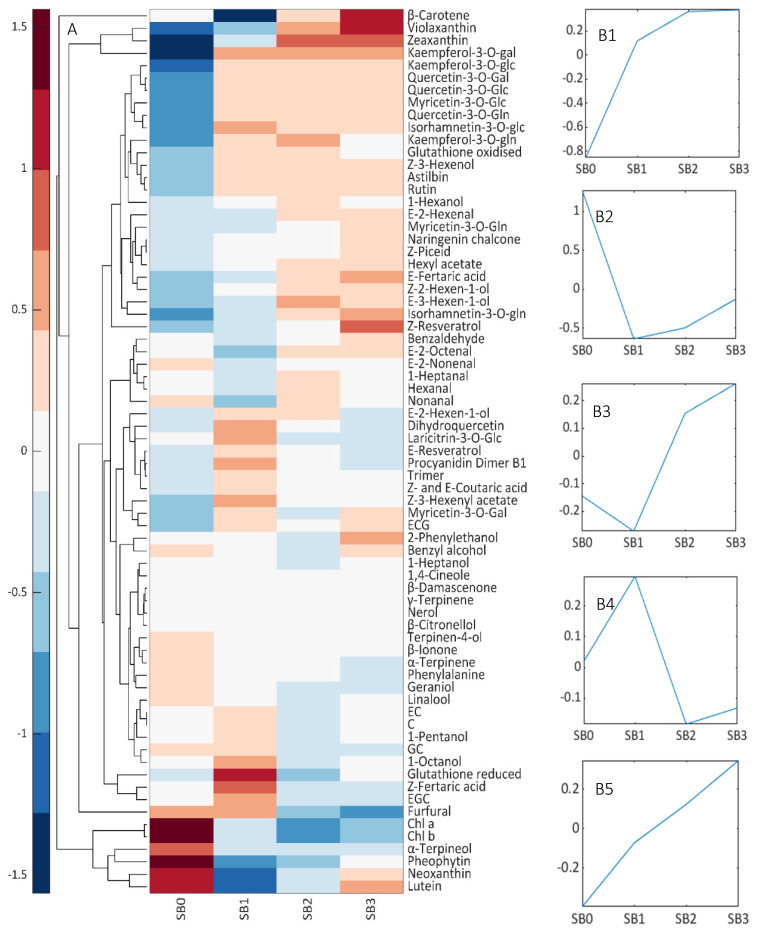
Impact of sunburn browning damage on carotenoid, polyphenol and free volatile compound concentration. (**A**) The heatmap represents metabolite concentrations at each damage level (SB0–SB3). Cell colours indicate relative concentrations for each SB level, with blue corresponding to the minimum and red to the maximum values. (**B1**–**B5**) Metabolite evolution pattern according to damage level. Variables in each group are listed in Supplementary Table S5. ECG, (−)-Epicatechin gallate; EC, (−)-Epicatechin; C, (+)-Catechin; GC, (+)-Gallocatechin; EGC, (−)-Epigallocatechin.

K-means clustering was used to investigate how compound concentration changed as SB damage increased. Five groups were evident (Figure 6, Supplementary Tables S4 and S5): Group 1 (G1; N = 18) was dominated by the flavonoids, violaxanthin and zeaxanthin, all of which increased rapidly between SB0 and 1 and remained high thereafter. G2 compounds (N = 6; neoxanthin, lutein, Chl *a* and *b*, pheophytin and α-terpineol) were rapidly degraded between SB0 and 1. G3 (N = 11) compounds β-carotene, aldehydes, 1,4-cineole, 1-hexanol and 2-phenylethanol initially saw their concentration decrease slightly between SB0 and 1 but increased as damage worsened. G4 (N = 30) consisted mainly of terpenes like linalool and α-terpinene, alcohols, reduced glutathione, *E*-resveratrol and flavan-3-ols, which increased in SB1 samples and decreased with subsequent damage. G5 (N = 6) was composed mainly of polyphenols such as M-3-*O*-gln, *Z*-resveratrol and *Z*-piceid, which increased steadily in concentration as SB damage increased.

## 4. Discussion

### 4.1. Excessive Light Promotes Changes to Berry Composition

Excessive light elicits a cascade of reactions from the exposed berry tissue that is not limited to a single group of compounds, but several types of compounds act simultaneously as UV- and PAR-screens, and as ROS scavengers. Zeaxanthin concentrations increased rapidly to counter higher light exposures in the defoliated treatments and remained higher in exposed berries (ED and LD) than in ND until harvest (Figure 1, Figure 2 and Figure 3). However, levels attained in LD were not as high as those in ED, probably due to a lower capacity to produce these compounds at and after véraison. As the berry ripens, the chloroplasts are dismantled and its ability to produce carotenoids to counter higher light intensities is progressively lost [12,28]. In situations of prolonged stress such as those undergone by the berries from the defoliated treatments, a second xanthophyll cycle—the lutein–lutein epoxide cycle—with lower relaxation rates is also activated [8]. A significant increase in lutein was observed in ED berries, although a similar increase was not observed in LD samples. The concentration of other important antioxidants such as β-carotene and neoxanthin followed similar trends as that of lutein, increasing significantly after defoliation in ED berries, but not in LD (Figure 3).

PP, and in particular flavonoids, were amongst the most affected compounds by higher levels of radiation, although a more pronounced impact was seen in ED than in LD (Figure 1, Figure 2 and Figure 3). The fruit environment strongly regulates the production of flavonoids [13], and early exposure has been found to have a greater impact on their concentrations than treatments performed at or after véraison [14,29]. Amongst these, Q and K appeared as the most reactive, as observed previously [30,31]. Likewise, flavan-3-ols (procyanidin B1, catechin and epicatechin) were always higher in ED berries, as observed in a recent study [29], and dehydroxylated subunits (catechin, epicatechin and epicatechin gallate) were favoured over B-ring trihydroxylated subunits (epigallocatechin) in ED and LD. Hydroxycinnamic acids have also been implicated in the plant’s defence against UV radiation [32]. These compounds are mainly synthesized during the early stages of development, and steadily decrease until harvest. As such, only interventions performed very close to the end of flowering had an effect on these metabolites and their precursors, and higher concentrations of most of these compounds were found in ED, particularly at BAL [33]. *p*-Coumaric acid is a precursor to many flavonoids [32], and a higher concentration of this compound can be related to higher amounts of flavonoids as confirmed by the strong positive correlation values obtained (r^2^ > 0.7; *p* < 0.001). Phenylalanine, a precursor in the phenylpropanoid pathway [33], exhibited very strong negative correlations with all of these compounds (r^2^ > 0.9; *p* < 0.001). Stilbenes such as piceid and resveratrol are well known grape antioxidants involved in constitutive and inducible plant defence responses [13,33], and higher concentrations of *Z*- and *E*-resveratrol, and *Z*-piceid were observed in the exposed samples than in the shaded ones.

The role of FV in light screening has received less attention than CAR and PP. However, these compounds are mainly located in the epidermis of berries and, together with their related enzymes, have been shown to respond to an altered light environment [12,13,34]. Examination of the XA loadings showed that several FV compounds were contributing to the separation of treatments at BAL. Results were varied according to vineyard location however, and FV compounds played a lesser role at CUM. As observed previously [12,35], some monoterpenes, namely, linalool, were higher in the exposed samples by harvest at both vineyards when compared with ND. α-Terpinene, terpinene-4-ol, α-terpineol, β-citronellol and nerol were also significantly different between treatments at BAL; while 1,4-cineole and geraniol were significantly different between treatments at CUM. Contrary to previous findings [12], the biggest effect on these compounds observed in this study seemed to stem from temperature rather than light exposure. However, radiation and temperature are strongly interrelated: higher radiation (caused by defoliation) can increase fruit surface temperature by up to 15 °C [30]. Variety-specific differences cannot be ruled out either: Chardonnay is only a moderate terpene producer [36] and these biosynthetic pathways might be less reactive in this grape variety. Other important groups that were significantly affected by defoliation but have received less attention with regards to abiotic stress responses in grapes were the C_6_ compounds. C_6_ compounds are abundant in grapes and are formed through the oxidative cleavage of polyunsaturated fatty acids (PUFAs) as part of the lipoxygenase–hydroperoxide lyase (LOX) pathway. ROS accumulation triggers PUFA formation and expression of the associated genes, which has been observed to increase C_6_ aldehyde and alcohol content in white grapes under ozone-induced stress conditions [37]. During stress events, plants remodel membrane fluidity, release large amounts of α-linolenic acid from membrane lipids, and upregulate LOX activity leading to higher C_6_ compound formation [38]. As observed by He et al. [10], ED depressed the synthesis of these compounds during the early stages of berry development, which suggests that in spite of higher light and stress conditions, at this stage, any incurred cell membrane damage was very limited.

### 4.2. Heat Triggered FV Biosynthesis and the Degradation of CAR and PP Metabolites

Temperature affects the speed of berry development, sugar accumulation and acid degradation [39] as was observed at CUM where berries ripened faster and had lower acidity levels regardless of defoliation treatment. Temperature also affects many other physiological processes and exacerbates photooxidative damage by multiplying the sources of stress [14]. Heat targets the photosynthetic system, impairs electron transport activity and increases anaerobic respiration and ROS accumulation. Under extreme conditions, as experienced at CUM, high temperatures cause cell death and SN. Furthermore, elevated heat alters the regulation of major metabolic pathways and the expression of genes involved in all levels of plant physiology, and increases the degradation of certain secondary metabolites [2]. Comparison of ND samples at véraison and harvest at both sites revealed significant differences for a range of CAR, FV and PP compounds (Table 1), and allowed us to delve deeper into the single effects of temperature rather than the combined effects of light and temperature.

A series of coping mechanisms such as the activation of the xanthophyll cycle (manifesting as significantly higher levels of zeaxanthin) and higher production of antioxidants such as β-carotene and reduced glutathione were detected as a consequence of the higher temperatures at CUM. All analysed flavonoids were higher in CUM than in BAL at véraison, although many of these differences had disappeared by harvest. Temperatures above 35 °C negatively affect flavonoid biosynthetic pathways and increase flavonoid degradation [2] as occurred in CUM, where a marked degradation of laricitrin, myricetin and isorhamnetin was observed between véraison and harvest. Although other authors have not observed differences in flavonoid concentration due to higher temperatures [29,40], the temperatures studied by these authors were limited to a maximum of 30 °C. Significantly higher temperatures (>45 °C) are required to see changes in the concentration of these compounds [22], akin to those experienced by CUM berries at véraison. High temperatures also had a significant effect over the berries’ redox balance, and higher concentrations of reduced glutathione were observed at CUM, probably to counter further oxidations and ROS. The influence of temperature on flavan-3-ol synthesis is not well understood to date, with authors showing contrasting results. This is also the case for hydroxycinnamic acids, where most studies have concentrated on the influence of light [22]. However, higher temperatures did seem to affect the concentration of these compounds in the present study, as evidenced by the overall higher amounts of these compounds at CUM than at BAL by harvest.

Amongst other effects, heat stress increases membrane fluidity. Volatile isoprenoids such as isoprene and the monoterpenes possess antioxidant properties, and although their specific mode of action still needs to be confirmed, some authors postulate that they physically stabilize membrane hydrophobic interactions by partitioning into the centre of phospholipid membranes [41]. Furthermore, the emission of monoterpenes (e.g., α-terpinene) quantitatively relates to stress severity, as larger increases in concentration have been observed at higher (>46 °C) than at more moderate temperatures (~30 °C) [42,43]. However, these studies have only delved into the emission of these compounds rather than their concentrations in intact organs, and more work is necessary to disentangle both effects. Since enzymes have different optimal temperatures, it is reasonable to assume that this behaviour is molecule-dependent and different chemical moieties would respond differently to different heat stress levels. As such, acyclic terpenes such as β-citronellol, linalool, geraniol and nerol were actually more abundant under the more moderate conditions at BAL than at CUM at both véraison and harvest, while the cyclic terpenes 1,4-cineole, α-terpineol, terpinene-4-ol and γ-terpinene were more abundant in CUM at véraison. Furthermore, recovery of enzymatic activity differs greatly according to temperature. A study on the release of mono- and sesquiterpenes from tomatoes [43] showed that β-phellandrene synthase required 24 h to recover initial activity levels after being treated at 37–41 °C during 5 min, and never recovered when treated at >46 °C. Despite higher concentrations at véraison, only 1,4-cineole continued to be more abundant at CUM than at BAL by harvest. This was to be expected as prolonged excessive temperatures not only impair correct enzymatic activity and thus volatile compound synthesis, but also increase their degradation and volatilization. Although studies on volatile compound emission under abiotic stress have mostly focused on isoprene and the monoterpenes, C_6_ compounds are characteristic plant stress volatiles that are quickly released in response to stress. Further, hexanal is the most abundant volatile compound in berries. Concentrations of hexanal and *E*-2-hexenal were higher in CUM berries than in BAL berries.

The 16 January sampling coincided with the second heatwave of the season (Figure 4), triggering compositional changes in berries across all treatments at both vineyards. This heatwave resulted in a spike in the concentrations of many compounds, particularly FV, that point to an adaptation effort by berries to the increased stress situation. This, however, needs to be confirmed by further experiments. The main compounds displaying this behaviour were all C_6_ compounds. Release of these compounds in plants is highest at temperatures >50 °C [44], as observed at CUM during the January heatwave. Higher levels of these compounds under these conditions also point to increased levels of cell membrane breakdown, coinciding with the higher levels of sunburn damage observed at this location.

### 4.3. Ripening Trumps All

The main differences amongst samples were accounted by ripening stage (factor XB), although it was possible to note similar degradation trends between ripening and during photooxidative damage [45]. Grape development follows a tightly coordinated program that is usually separated into three major stages dictated by berry size growth and sugar accumulation but also by the biosynthesis of specific secondary metabolites. Most gene expression is time-dependent [14], and earlier exposure affected more stress-related genes than in berries exposed at véraison, which allows for more adaptation mechanisms to be activated. Carotenoids, chlorophylls, the flavan-3-ols, hydroxycinnamic and organic acids are synthesized during the first stage, and as berries further developed, a marked degradation of these compounds, regardless of treatment, was observed. Véraison marks the start of stage III and berry softening and is characterized by an oxidative burst that causes an overexcitation of the antioxidant systems akin to that provoked by photooxidative stress [45]. A consistent increase in aldehydes, 2-ethyl-1-hexanol and some terpenes, as well as the flavonoids Q and K was observed post-véraison, while the concentrations of terpinene-4-ol, α-terpineol, β-citronellol, geraniol, α- and β-ionone, β-damascenone, benzyl alcohol and phenylethanol were all highest at véraison and decreased thereafter as previously observed [46]. Likewise, the flavan-3-ols and the hydroxycinnamic acids were all lower at harvest. Overall, a significant interaction between treatment and timing was observed at both vineyards that denotes the fact that defoliation treatments performed at different developmental stages had different outcomes. In general, by véraison, the berry’s capacity to produce important antioxidants and light screen compounds decreases, which makes it more vulnerable to sunburn damage when berries are exposed to higher radiation levels at this later stage. As observed from the results of this study as well as those by others [12,14,29,47], earlier defoliations have a more pronounced and positive effect on CAR and PP production, and are ultimately more effective at protecting berries from the negative consequences of excessive sunlight.

### 4.4. Sunburn Damage

The year 2019 remains to date one of the hottest on record in Australia [48]. The high temperatures experienced in summer that year, together with an ongoing drought, created the perfect conditions for sunburn damage to manifest in sun-exposed grapes. Sunburn damage appears to be worse when unacclimated organs are suddenly exposed to excessive radiation and temperature, as occurred to LD berries at both vineyards (Table 2, Figure 5). Despite accumulating overall more degree days and radiation than those of any other treatment, ED berries were first exposed when temperatures were still relatively mild in late spring (especially at BAL, Figure 4) and therefore developed adaptive mechanisms that limited sunburn susceptibility [8,29], while LD berries were exposed when air temperatures were at their harshest, which impaired many biosynthetic pathways and the production of photoprotectants. In the particular case of CUM, LD immediately preceded the January 15th heatwave, which reached maximum berry temperatures of ~50 °C. Temperature has a major effect on the appearance and severity of sunburn damage [2,6,17], and under the particular conditions at CUM (Figure 4), SN of exposed LD berries immediately ensued. Overall, a lower level of sunburn damage and proportion of damaged berries were observed across the different treatments at BAL, where due to higher elevation, maximum air and berry temperatures were lower than at CUM.

Adaptation strategies such as the production of carotenoids and management of photosynthetic pigments [6,8,45] were apparent in the samples analysed as SB damage increased. Foremost, increasing SB damage caused the preferential destruction of Chl *b*, as has been observed by others [18], and the increase of β-carotene, which consequently affected the Chl *a*/*b* and CAR/Chl ratios. Chl *b* underwent a stronger degradation than did Chl *a*, which suggests a light acclimation response involving the reduction in the amount of light-harvesting complex II [3,7]. An upregulation of zeaxanthin and violaxanthin production was also evident as an effort to dissipate excess energy through NPQ and activation of the xanthophyll cycle [7,8,16]. The upward trend of G2 compounds demonstrated that there was no limitation to the production of these compounds, and that sufficient precursors, namely, β-carotene, were available.

Activation of other antioxidant mechanisms was also apparent, particularly when SB damage was mild. Glutathione is an important antioxidant, part of the ascorbate–glutathione cycle, which counteracts sunburn damage by maintaining tissue redox status and scavenging harmful ROS and hydrogen peroxide [2]. An initial increase in the production of reduced glutathione between SB0 and SB1 was observed, followed by lower levels of this compound and a rise in the oxidized form of glutathione (G1) and the glutathione reduced/oxidized ratio as damage increased. This ratio was lowest in SB1 samples, suggesting a higher redox capacity, which peaked in SB2 samples. An initial rise in *E*-resveratrol was also observed, but only between SB0 and SB1. Reportedly, resveratrol enhances antioxidant activities in transgenic plants that overexpress this metabolite, and aids in membrane stabilization in UV-C-irradiated plants [49,50]. Linalool and several other terpenes (G4) also markedly increased between SB0 and SB1 and decreased thereafter, indicating that all these compounds may act as transient photoprotective systems only during the initial stages of sunburn damage, and that higher SB could have important consequences for the sensory profile of any ensuing wine. Although FV are important compounds for wine quality, no previous study has examined the consequences of SB on these compounds.

Flavonoids (G1) are important antioxidants, and their concentrations increased rapidly between SB0 and SB1, and remained stable regardless of increasing sunburn damage. The biggest changes were observed with respect to Q- and K-3-*O*-glc, which exhibited a 5- and 10-fold rise in concentration, respectively. Quercetin derivatives chelate Cu and Fe ions to inhibit the generation of ROS, and the accumulation of Q-3-*O*-glc contributes to the brown appearance of SB fruit that was noticeable even in SB1 samples [6]. *Z*-resveratrol, piceid (the 3-*O*-β-D-glu derivative of resveratrol), *E*-2-hexenal, 1-hexanol and 2-phenylethanol (G3 and G5) also showed a sustained increase. Remarkably, G1 and G3 also contained an important number of C_6_ compounds that could be a result of membrane denaturation in grapes with higher levels of sunburn damage [37].

Contrary to previous observations [7], higher sunburn levels resulted in a sharp fall in flavan-3-ol content despite an initial increase in concentration. This same behaviour was observed in selected hydroxycinnamic acids. Some terpenes and norisoprenoids (G4, e.g., α-terpinene, terpinene-4-ol, α-terpineol, β-ionone and β-damascenone) sustained a sharp fall in concentration even with mild sunburn damage, possibly as a result of the higher temperatures sunburn-damaged berries were exposed to.

## 5. Conclusions

This study has shown how adaption mechanisms change in grapes according to developmental stage, and how grapes utilize different sunscreen compounds simultaneously in a coordinated effort to protect themselves from photooxidative and sunburn damage. Due to a combination of high humidity and rain during the ripening seasons, many cool-climate regions still rely heavily on practices such as leaf removal that decrease shading in the bunch zone and thus increase the risk of sunburn. However, as the risk of sunburn increases with climate change even in regions historically considered as having cool climates, adaptation of this important practice is required. Our results show that an earlier defoliation would lead to a better acclimation of grapes and thus less sunburn damage and overall quality loss. However, high radiation levels and higher temperature can have antagonistic effects. Whilst higher carotenoids, flavonoids and selected free volatiles were observed in defoliated treatments, higher temperatures appear to have been detrimental to the adaptation of berries by limiting production of these compounds and also increasing their rate of degradation, which further stresses the need to perform activities such as defoliation when temperatures are still mild. Finally, the compositional changes in secondary metabolites experienced by berries as they underwent sunburn damage were also outlined. The behaviour of important metabolites for wine quality, such as the C_6_ compounds, linalool and other aroma compounds, during the development of sunburn damage were described for the first time.

## Figures and Tables

**Table 1:** Composition (CAR, PP and FV) of nondefoliated samples from BAL and CUM at véraison and harvest.

		Véraison			Harvest		
		BAL	CUM	*p*	BAL	CUM	*p*
**CAR ^1^**	Carotenoids						
	Neoxanthin	0.68 ± 0.06	0.76 ± 0.18	ns ^2^	0.29 ± 0.04	0.23 ± 0.06	ns
	Violaxanthin	0.60 ± 0.04	0.77 ± 0.11	ns	0.39 ± 0.08	0.30 ± 0.10	ns
	Zeaxanthin	0.50 ± 0.04	1.2 ± 0.2	*	0.47 ± 0.06	2.0 ± 0.6	*
	Lutein	8.6 ± 0.2	11 ± 2	ns	4.0 ± 0.7	4.0 ± 1.0	ns
	β-Carotene	2.6 ± 0.5	4.9 ± 1.2	*	2.1 ± 0.3	2.7 ± 0.5	ns
	Chlorophylls						
	Chlorophyll *a*	13 ± 0.4	19 ± 0.7	**	4.4 ± 0.9	6.3 ± 2.6	ns
	Chlorophyll *b*	6.6 ± 0.7	7.2 ± 1.5	ns	2.9 ± 0.4	1.8 ± 0.5	*
**PP**	Flavonoids						
	Quercetin 3-*O*-glc (µg/g) ^3^	11 ± 2	31 ± 5	*	48 ± 8	41 ± 10	ns
	Quercetin 3-*O*-gal (µg/g)	1.0 ± 0.3	4.1 ± 0.6	*	8.1 ± 1.3	6.5 ± 1.4	ns
	Quercetin 3-*O*-gln (µg/g)	21 ± 5	32 ± 5	*	7.7 ± 1.4	10 ± 1	ns
	Laricitrin 3-*O*-glc	9.3 ± 0.8	21 ± 0.2	***	2.8 ± 0.2	7.6 ± 1.7	*
	Myricetin 3-*O*-glc	78 ± 0.6	165 ± 35	*	22 ± 3	80 ± 14	*
	Myricetin 3-*O*-gal	7.5 ± 0.9	17 ± 4	**	3.7 ± 0.3	6.3 ± 2.0	ns
	Myricetin 3-*O*-gln	48 ± 7	123 ± 27	*	24 ± 2	28 ± 4	ns
	Isorhamnetin 3-*O*-glc	229 ± 49	321 ± 41	ns	95 ± 13	112 ± 20	ns
	Isorhamnetin 3-*O*-gln	4.1 ± 0.4	13 ± 3	*	9.2 ± 2.1	17 ± 3	*
	Kaempferol 3-*O*-glc (µg/g)	0.20 ± 0.06	0.64 ± 0.07	**	7.3 ± 1.5	6.5 ± 0.9	ns
	Kaempferol 3-*O*-gal (µg/g)	0.07 ± 0.02	0.19 ± 0.00	**	1.6 ± 0.4	1.4 ± 0.3	ns
	Kaempferol 3-*O*-gln (µg/g)	0.18 ± 0.03	0.31 ± 0.03	*	0.29 ± 0.02	0.29 ± 0.05	ns
	Astilbin (µg/g)	0.13 ± 0.02	0.31 ± 0.05	*	1.3 ± 0.09	0.84 ± 0.11	*
	Dihydroquercetin	122 ± 9	42 ± 6	***	38 ± 4	17 ± 3	**
	Rutin	55 ± 3	74 ± 10	*	200 ± 8	149 ± 20	*
	Procyanidin Dimer B1 (µg/g)	2.0 ± 0.1	1.5 ± 0.1	*	0.77 ± 0.15	0.98 ± 0.11	ns
	Flavan-3-ols						
	(+)-Gallocatechin (µg/g)	10 ± 1	11 ± 2	ns	2.6 ± 0.3	4.6 ± 0.8	*
	(−)-Epigallocatechin (µg/g)	0.43 ± 0.08	0.49 ± 0.06	ns	0.26 ± 0.03	0.95 ± 0.11	**
	(+)-Catechin (µg/g)	19 ± 2	12 ± 1	*	5.9 ± 1.0	4.2 ± 0.7	ns
	(−)-Epicatechin (µg/g)	15 ± 1	9.3 ± 1.2	*	4.7 ± 0.8	3.5 ± 0.5	ns
	(−)-Epicatechin gallate	24 ± 5	15 ± 2	*	10 ± 2	12 ± 1	ns
	Stilbenoids and chalcones						
	*Z*-Resveratrol	74 ± 6	63 ± 11	ns	140 ± 11	47 ± 3	***
	*E*-Resveratrol (µg/g)	4.7 ± 0.3	3.6 ± 0.3	*	1.8 ± 0.3	2.3 ± 0.3	ns
	Trimer	140 ± 10	60 ± 7	**	50 ± 7	39 ± 3	ns
	*Z*-Piceid	2.3 ± 0.09	2.2 ± 0.4	ns	5.4 ± 0.4	3.7 ± 0.5	*
	Naringenin chalcone	0.82 ± 0.05	0.73 ± 0.04	ns	2.5 ± 0.2	1.5 ± 0.3	*
	Amino Acids						
	Tyrosine	44 ± 7	71 ± 4	*	236 ± 41	446 ± 38	*
	Tryptophan	0.73 ± 0.03	0.82 ± 0.1	ns	0.48 ± 0.05	0.74 ± 0.11	*
	Phenylalanine (µg/g)	1.1 ± 0.1	1.2 ± 0.1	ns	3.0 ± 0.4	3.8 ± 0.3	ns
	Glutathione (reduced)	171 ± 40	723 ± 42	***	595 ± 141	1641 ± 3	***
	Glutathione (oxidized)	9.3 ± 0.8	2.6 ± 0.3	***	3.6 ± 1.3	1.9 ± 0.2	ns
	Hydroxycinnamic Acids						
	*Z-*Caftaric acid (µg/g)	204 ± 13	202 ± 13	ns	48 ± 2	81 ± 8	*
	*E*-Caftaric acid (µg/g)	124 ± 12	119 ± 4	ns	40 ± 3	62 ± 3	**
	*p-*Coumaric acid	2.1 ± 0.3	1.9 ± 0.4	ns	0.46 ± 0.11	1.1 ± 0.0	*
	*Z-* and *E*-Coutaric acid (µg/g)	757 ± 48	689 ± 62	ns	166 ± 23	336 ± 36	*
	*Z*-Fertaric acid	873 ± 63	765 ± 156	ns	15 ± 4	71 ± 13	*
	*E*-Fertaric acid (µg/g)	17 ± 2	16 ± 2	ns	7.1 ± 0.6	17 ± 2	*
	Syringic acid	230 ± 6	296 ± 10	**	113 ± 8	220 ± 27	*
**FV**	Aldehydes						
	Hexanal (µg/g)	6.3 ± 0.7	10 ± 1	*	34 ± 1	49 ± 2	***
	*E*-2-Hexenal (µg/g)	1.8 ± 0.1	3.6 ± 0.5	*	11 ± 0	20 ± 1	***
	1-Heptanal	4.8 ± 0.7	6.4 ± 0.3	*	28 ± 3	52 ± 5	*
	*E*-2-Octenal	2.1 ± 0.2	3.1 ± 0.4	*	13 ± 1	16 ± 3	ns
	Nonanal	5.8 ± 0.4	5.6 ± 0.1	ns	40 ± 8	93 ± 13	*
	*E*-2-Nonenal	14 ± 0	14 ± 0	*	15 ± 0	14 ± 0	**
	Benzaldehyde	0.79 ± 0.09	0.73 ± 0.1	ns	5.7 ± 0.2	3.4 ± 0.4	**
	Furfural	45 ± 0.1	190 ± 58	*	128 ± 24	237 ± 7	*
	Isoprenoids						
	1,4-Cineole	0.29 ± 0.06	0.90 ± 0.05	**	0.048 ± 0.007	0.065 ± 0.005	*
	α-Terpinene	0.60 ± 0.19	0.80 ± 0.02	ns	0.17 ± 0.04	0.25 ± 0.06	ns
	γ-Terpinene	0.066 ± 0.008	0.082 ± 0.005	*	0.027 ± 0.002	0.026 ± 0.006	ns
	Terpinen-4-ol	0.38 ± 0.01	1.1 ± 0.1	**	0.17 ± 0.03	0.12 ± 0.03	ns
	α-Terpineol	7.1 ± 0.9	14 ± 3	*	4.3 ± 0.9	2.1 ± 0.3	**
	β-Citronellol	0.40 ± 0.05	0.31 ± 0.07	ns	0.045 ± 0.001	0.025 ± 0.001	*
	Linalool	4.2 ± 0.2	4.0 ± 0.1	ns	5.7 ± 0.0	5.1 ± 0.3	*
	Nerol	0.048 ± 0.007	0.033 ± 0.003	*	0.072 ± 0.003	0.043 ± 0.009	*
	Geraniol	2.3 ± 0.5	0.85 ± 0.03	*	0.88 ± 0.10	0.51 ± 0.00	*
	C_13_-apocarotenoids						
	β-Damascenone	0.52 ± 0.00	0.47 ± 0.08	ns	0.024 ± 0.003	0.016 ± 0.002	*
	α-Ionone	0.025 ± 0.001	0.044 ± 0.001	*	0.018 ± 0.001	0.021 ± 0.001	*
	β-Ionone	1.4 ± 0.3	1.2 ± 0.3	ns	0.41 ± 0.02	0.30 ±0.06	*
	Alcohols						
	1-Pentanol	24 ± 1	36 ± 10	ns	31 ± 1	41 ± 7	ns
	1-Hexanol	25 ± 5	11 ± 0.7	*	64 ± 13	54 ± 5	ns
	*E*-2-Hexenol	19 ± 2	7.4 ± 0.3	**	38 ± 4	21 ± 1	*
	*Z*-2-Hexenol	0.35 ± 0.03	0.22 ± 0.02	*	0.36 ± 0.07	0.11 ± 0.01	*
	*E*-3-Hexenol	1.9 ± 0.4	1.4 ± 0.4	ns	1.2 ± 0.0	1.4 ± 0.3	ns
	*Z*-3-Hexenol	48 ± 10	18 ± 2	*	16 ± 1	3.2 ± 0.2	***
	2-Ethyl-1-hexanol	4.3 ± 0.6	3.6 ± 0.5	ns	6.9 ± 0.9	5.8 ± 0.5	ns
	1-Heptanol	1.5 ± 0.1	1.3 ± 0.1	*	2.1 ± 0.4	3.3 ± 0.5	*
	1-Octanol	1.6 ± 0.2	1.5 ± 0.2	ns	13 ± 0	7.3 ± 1.0	**
	Benzyl alcohol	2.9 ± 0.2	1.6 ± 0.2	*	1.2 ± 0.1	1.1 ± 0.1	ns
	Phenylethanol	15 ± 1	8.9 ± 1.8	*	4.4 ± 0.0	3.1 ± 0.1	***
	Others						
	Hexyl acetate	0.24 ± 0.04	0.23 ± 0.05	ns	0.42 ± 0.12	0.077 ± 0.015	*
	*Z*-3-Hexenyl acetate	8.1 ± 0.8	2.9 ± 0.5	**	1.6 ± 0.1	0.26 ± 0.04	***
	Hexanoic acid	93 ± 21	203 ± 8	*	72 ± 17	82 ±8	ns

^1^ Dataset: CAR, carotenoids; PP, polyphenols; FV, free volatiles. ^2^ Indicates level of significance according to paired t-test comparison of means at a 95% confidence level: ns, nonsignificant; * *p* < 0.05; ** *p* < 0.001; *** *p* < 0.0001. ^3^ All quantities expressed as ng/g unless stated. Glc, glucoside; gal, galactoside; gln, gluconoride.

**Table 2:** Percentage (%) of berries exhibiting sunburn damage in BAL and CUM bunches at harvest.

Vineyard	Damage Level	ND ^1^	ED	LD
BAL	SB1 ^2^	10 ± 0 a ^3^	28 ± 15 c	19 ± 8 b
	SB2	2 ± 4 a	9 ± 9 ab	26 ± 10 c
	SN	4 ± 6	3 ± 5	4 ± 3
CUM	SB1	3 ± 4 a	8 ± 5 b	8 ± 6 b
	SB2	3 ± 4	11 ± 9	9 ± 9
	SN	4 ± 6 a	8 ± 3 a	22 ± 14 b

^1^ Designates treatments: no defoliation, ND; early defoliation, ED; late defoliation, LD. ^2^ Designates type and intensity of damage: SB, sunburn browning ranging from level 1 to 2; SN, sunburn necrosis. ^3^ Different letters across a row designate significant differences (α = 0.05) between different treatments within a vineyard, according to Tukey’s HSD.

## Data Availability

The data supporting the findings of this study are available from the corresponding author, Joanna M. Gambetta, upon request.

## Supplementary Materials

Additional experimental details, materials and methods and the following are available online at https://www.mdpi.com/article/10.3390/biom12010042/s1. Table S1: Vineyard characteristics and key trial dates. Table S2: Mean values of berry weight (n = 50), pH, titratable acidity (TA), total soluble solids (TSS) and yeast assimilable nitrogen (YAN) for both sites (BAL and CUM) and all treatments at harvest. Table S3: Monthly total radiation, average daily maximum solar radiation, average temperature and rainfall from December 2018 until January 2019. Table S4: Chemical composition of berries with varying levels of sunburn brown damage (SB0–SB3). Table S5: Data Blocks, ASCA Variable indices and K-means clustering.
