# Supplementary material for: Secondary Metabolites Coordinately Protect Grapes from Excessive Light and Sunburn Damage during Development
Source: Biomolecules. 2021 Dec 28;12(1):42. doi: 10.3390/biom12010042 (PMC8773587; doi:10.3390/biom12010042)
Supplement: Supplementary file 1 [file biomolecules-12-00042-s001.zip › biomolecules-1461074-supplementary.pdf]

SUPPLEMENTARY INFORMATION FOR

Secondary metabolites coordinately protect grapes from excessive light and sunburn damage during development

Joanna M. Gambetta[a,b,1*], Valentina Romat[a], Leigh M. Schmidtke[a], Bruno Holzapfel[a,c]

[a]School of Agricultural, Environmental and Veterinary Sciences, National Wine and Grape Industry Centre, Charles Sturt University, Locked Bag 588, Wagga Wagga, NSW 2678, Australia

[b]Waite Research Institute, School of Agriculture, Food and Wine, University of Adelaide, PMB1, Glen Osmond, South Australia 5064, Australia

[c]Wagga Wagga Agriculture Institute, New South Wales Department of Primary Industries, Wagga Wagga, NSW 2650, Australia

*Correspondence: joanna.gambetta@adelaide.edu.au

[1]Present Address: CSIRO Agriculture and Food, 671 Sneydes Road, Werribee, VIC 3030, Australia

5. Experimental

5.1 Climatic monitoring and vineyard measurements

Growing degree day (DD) for each site were calculated between the October 1st and April 30th using 10 °C as base temperature. Leaf area (m^2, LA) for every treatment was calculated shortly after each defoliation treatment was implemented. Ten representative shoots were collected from every row and transported to the lab where the number of leaves per shoot was counted and their area measured using a LLI-3100C leaf area meter (LI-COR, Lincoln, Nebraska, USA). LA per plant was estimated by multiplying unit leaf area, average number of leaves per shoot and number of shoots per plant.

Midday stem water potential (SWP) determinations were carried out every two weeks between 12h00 and 14h00 with a single leaf removed per vine per measurement as described by Choné et al. (2001) and Rossouw et al. (2017) (Choné et al., 2001; Rossouw et al., 2017). Measurements were performed using a pressure chamber (Model 1000, PMS instruments, Albany, Oregon, USA).

Yield per plant at harvest was estimated by counting and weighing all bunches in each plant.

5.5 Chemical analyses

5.5.1 Reagents

Deuterated internal standards d3-linalool, d13-1-hexanol, d12-hexanal were supplied by CDN Isotopes (Point-139 Claire, Quebec, CN). Lutein (94%), zeaxanthin (97%), violaxanthin (95%) and neoxanthin (97%) were obtained from CaroteNature GmbH (Lupsingen, Switzerland). Sodium chloride (analytical grade) was provided by JT Baker (Phillipsburg, New Jersey, USA) and water was obtained from a Milli-Q purification system (Millipore, North Ryde, NSW, Australia).

5.5.2 Basic grape chemistry

Total soluble solids (TSS) were measured using a digital portable refractometer (PR-101, Atago, Tokyo, Japan) and titratable acidity (TA) and pH were measured using an automatic titrator (Metrohm Fully Automated 59 Place Titrando System, Metrohm AG, Herisau, Switzerland) to an end point of pH 8.2.

5.5.5 Carotenoid and chlorophyll extraction and UPLC-DAD analysis

Samples for carotenoid and chlorophyll analysis were first deseeded and ground under liquid nitrogen using an A11 basic analytical mill (IKA, Selangor, Malaysia). Two mL of a mix of methanol, chloroform and water (2:2:1) were added to 1 g of frozen grape berry powder, and 20 µL trans-β-Apo-8'-carotenal (25 µg/L in ethyl acetate) was used as internal standard. Samples were then briefly vortexed, extracted for 15 min with a Ratek rotary mixer (RSM7DC, Ratek Instruments Pty Ltd, Boronia, Victoria, Australia) and centrifuged. The chloroform layer was then collected and evaporated to dryness under a stream of nitrogen after addition of 20 µL of a solution of TEA (0.1% V/V in chloroform). Samples were resuspended with 100 µL ethyl acetate and 400 µL methanol immediately before injection. Standard stock solutions were prepared in ethyl acetate (carotenoids) or acetone (Chlorophyll a) and dilutions were made using ethyl acetate.

5.6 Statistical analyses

All blocks were pre-treated prior to ASCA as follows: CAR data was normalized using unit-variance scaling and PP and FV were mean-centered and Pareto-scaled. All blocks were then scaled by dividing

each matrix by its Frobenius' norm, and concatenated into a single megablock with 80 variables (Biancolillo et al., 2015). One thousand permutation rounds were performed to assess the statistical significance of each factor and their interaction.

Prior to MB-SO-PLS-LDA, variables that were not significantly different (according to 1-way ANOVA) between SB damage levels were removed from each block. All blocks (CAR, PP and FV) were then pre-treated as for ASCA, but they were not scaled. Block order and inclusion were determined by the combination yielding the best classification results after testing all possible combinations. The order determined was PP-FV. Optimal number of latent variables (LVs) was determined using a Mage Plot as the combination that yielded both the least RMSECV and lowest % classification error (Biancolillo et al., 2015).

References

Biancolillo, A., Måge, I., & Næs, T. (2015). Combining SO-PLS and linear discriminant analysis for multi-block classification. *Chemometrics and Intelligent Laboratory Systems*, *141*, 58–67. https://doi.org/10.1016/j.chemolab.2014.12.001

Choné, X., Van Leeuwen, C., Dubourdieu, D., & Gaudillère, J. P. (2001). Stem water potential is a sensitive indicator of grapevine water status. *Annals of Botany*, *87*(4), 477–483. https://doi.org/10.1006/anbo.2000.1361

Rossouw, G. C., Smith, J. P., Barril, C., Deloire, A., & Holzapfel, B. P. (2017). Carbohydrate distribution during berry ripening of potted grapevines: Impact of water availability and leaf-to-fruit ratio. *Scientia Horticulturae*, *216*, 215–225. https://doi.org/10.1016/j.scienta.2017.01.008

Table S1. Vineyard characteristics and key trial dates

		Vineyard	
		**BAL**	**CUM**
Location		33°16’03.9” S, 148°59’59.0” E	32°58’47.4” S, 148°57’42.8” E
Altitude		884 m.a.s.l.	612 m.a.s.l.
Date of plantation		1995 (grafted onto Shiraz)	2000 (own roots)
Defoliation Timing	ND[1]	Not performed	Not performed
	ED	6/12/2018 (9 DAF[2])	6/12/2018 (17 DAF)
	LD	23/1/2019 (56 DAF)	8/1/2019 (50 DAF)
Harvest		1/3/2019	4/2/2019

[1]Designates treatments: no defoliation, ND; early defoliation, ED; late defoliation, LD. [2] Days after flowering, DAF

Table S2. Mean and standard deviation values of bunch number, bunch weight, yield and leaf area (LA) per plant and LA per kg of fruit for both sites (BAL and CUM) at harvest.

**Vineyard**		**ND**[1]	**ED**	**LD**
BAL	No. of bunches/vine	36 ± 3	40 ± 2	42 ± 2
	Bunch weight (g)	125 ± 16	122 ± 16	125 ± 33
	Yield/plant (kg)	4.49 ± 0.78	4.84 ± 0.89	5.25 ± 1.31
	LA (m^2)	11.1 ± 3.3	9.1 ± 2.7	7.9 ±2.1
	LA/kg fruit (m^2/kg)	2.4 ± 0.4a[2]	1.8 ± 0.4a	1.4 ± 0.2b
CUM	No. of bunches/vine	50 ± 12	42 ± 6	51 ± 6
	Bunch weight (g)	93 ± 24	89 ± 25	87 ± 23
	Yield/plant (kg)	4.55 ± 1.15	3.85 ± 1.61	4.45 ± 1.16
	LA (m^2)	8.0 ± 0.6	6.0 ± 1.5	6.3 ± 2.0
	LA/kg fruit (m^2/kg)	1.8 ± 0.3	1.7 ± 1.1	1.4 ± 0.7

[1]Designates treatments: no defoliation, ND; early defoliation, ED; late defoliation, LD. [2]Different letters across a row designate significant differences between different treatments within a vineyard, according to Tukey's HSD.

Table S3. Monthly total radiation, average daily maximum solar radiation, average temperature and rainfall from December 2018 until January 2019

Vineyard	Month	Total Radiation (mol/m^2)	Average daily max. solar radiation (W/m^2)	Avg. temp (°C)	Rainfall (mm)
BAL	Dec	1241	667	21.9	91
	Jan	1590	663	26.0	68
	Feb	1378	635	20.9	48
	Growing Season	1403	655	22.9	69
CUM	Dec	1296	665	24.1	61
	Jan	1369	615	28.2	69
	Growing Season	1333	640	26.2	65

Table S4. Chemical composition of berries with varying levels of sunburn browning damage (SB0-SB3)

		SB0	***SB1***	***SB2***	***SB3***
**CAR**	Carotenoids				
	Neoxanthin	0.49 ± 0.04b[1]	0.39 ± 0.03a	0.43 ± 0.01ab	0.45 ± 0.03b
	Violaxanthin	0.73 ± 0.03a	0.88 ± 0.03b	1.1 ± 0.03c	1.2 ± 0.08c
	Zeaxanthin	0.29 ± 0.01a	1.5 ± 0.2b	2.6 ± 0.05c	2.8 ± 0.05c
	Lutein	7.6 ± 0.1b	6.4 ± 0.2a	6.9 ± 0.03ab	7.3 ± 0.5b
	β-Carotene	3.9 ± 0.2b	3.2 ± 0.2a	4.1 ± 0.2bc	4.6 ± 0.3c
	Chlorophylls				
	Chlorophyll a	10 ± 1c	6.0 ± 0.7b	4.2 ± 0.8a	4.9 ± 0.3ab
	Chlorophyll b	5.4 ± 0.6b	3.7 ± 0.05a	3.1 ± 0.1a	3.4 ± 0.4a
	Chlorophyll a/b	*2.0 ± 0.2*	*1.6 ± 0.2*	*1.4 ± 0.3*	*1.5 ± 0.1*
	β-*Carotene/Chlorophyll*	*0.73 ± 0.06*	*0.86 ± 0.04*	*1.3 ± 0.0*	*1.4 ± 0.1*
**PP**	Flavonoids				
	Quercetin 3-O-glc (µg/g)[2]	20 ± 1a	89 ± 9b	108 ± 5c	111 ± 4c
	Quercetin 3-O-gal (µg/g)	3.3 ± 0.3a	15 ± 1b	19 ± 1c	20 ± 2c
	Quercetin 3-O-gln (µg/g)	2.7 ± 0.2a	12 ± 2c	9 ± 1b	9 ± 1b
	Laricitrin 3-O-glc	2.4 ± 0.2a	4.5 ± 0.3b	2.2 ± 0.1a	2.2 ± 0.0a
	Myricetin 3-O-glc	12 ± 0.5a	34 ± 7b	33 ± 0.4b	42 ± 6b
	Myricetin 3-O-gal	3.0 ± 0.8a	7.2 ± 0.4b	4.2 ± 0.4a	6.2 ± 0.5b
	Myricetin 3-O-gln	20 ± 5a	22 ± 2a	26 ± 6a	33 ± 2b
	Isorhamnetin 3-O-glc	22 ± 0.2a	122 ± 5b	97 ± 21b	95 ± 20b
	Isorhamnetin 3-O-gln	3.3 ± 0.5a	9.7 ± 2.2b	22 ± 5c	26 ± 2c
	Kaempferol 3-O-glc (µg/g)	2.1 ± 0.2a	15 ± 3b	20 ± 0.9b	20 ± 1b
	Kaempferol 3-O-gal (µg/g)	0.08 ± 0.0a	4.3 ± 0.3b	5.3 ± 0.4c	5.3 ± 0.4c
	Kaempferol 3-O-gln (µg/g)	0.09 ± 0.01a	0.26 ± 0.02b	0.41 ± 0.05c	0.25 ± 0.01b
	Astilbin (µg/g)	0.94 ± 0.07a	1.6 ± 0.1b	1.8 ± 0.1b	1.7 ± 0.3b
	Dihydroquercetin	28 ± 0.4a	53 ± 12b	33 ± 2a	25 ± 5a
	Rutin (µg/g)	0.16 ± 0.01a	0.26 ± 0.02b	0.27 ± 0.01b	26 ± 0.03b
	Procyanidin Dimer B1 (µg/g)	0.82 ± 0.05a	1.32 ± 0.10b	0.96 ± 0.11a	0.86 ± 0.13a
	Flavan-3-ols				
	(+)-Gallocatechin (µg/g)	3.7 ± 0.1ab	4.6 ± 0.8b	2.5 ± 0.4a	2.8 ± 0.3a
	(-)-Epigallocatechin (µg/g)	0.47 ± 0.02b	0.78 ± 0.2c	0.27 ± 0.01a	0.31 ± 0.05a
	(+)-Catechin (µg/g)	7.5 ± 0.2ab	8.2 ± 0.7b	6.0 ± 0.7a	6.4 ± 0.9ab
	(-)-Epicatechin (µg/g)	6.1 ± 0.1ab	6.5 ± 0.5b	4.8 ± 0.6a	5.2 ± 0.7a
	(-)-Epicatechin gallate	7.6 ± 0.4a	17 ± 3c	12 ± 1b	18 ± 0.7c
	Stilbenoids and chalcones				
	Z-Resveratrol (µg/g)	0.15 ± 0.02a	0.18 ± 0.04a	0.49 ± 0.19b	0.78 ± 0.08c
	E-Resveratrol (µg/g)	2.0 ± 0.3a	3.1 ± 0.2b	2.3 ± 0.3a	2.1 ± 0.3a
	Trimer	48 ± 0.5a	75 ± 10b	56 ± 2a	52 ± 8a
	Z-Piceid	4.7 ± 0.2a	6.2 ± 0.3b	6.6 ± 0.1b	7.2 ± 0.8b
	Naringenin chalcone	2.1 ± 0.1a	2.8 ± 0.1b	3.0 ± 0.1b	3.3 ± 0.5b
	Amino Acids				

	Tyrosine (µg/g)	0.30 ± 0.03	0.31 ± 0.07	0.30 ± 0.01	0.26 ± 0.03
	Tryptophan	0.62 ± 0.05	0.62 ± 0.01	0.59 ± 0.08	0.57 ± 0.12
	Phenylalanine (µg/g)	4.1 ± 0.4b	3.5 ± 0.6ab	3.1 ± 0.1ab	2.6 ± 0.3a
	Glutathione reduced (µg/g)	0.35 ± 0.02ab	3.0 ± 0.08c	0.29 ± 0.11a	0.70 ± 0.18b
	Glutathione oxidised (ng/g)	2.6 ± 0.2a	7.3 ± 1.5b	5.3 ± 1.4b	5.6 ± 1.0b
	Hydroxycinnamic Acids				
	cis-Caftaric acid (µg/g)	66 ± 7	67 ± 8	65 ± 1	73 ± 5
	trans-Caftaric acid (µg/g)	51 ± 5	55 ± 11	44 ± 5	46 ± 7
	p-Coumaric acid	0.42 ± 0.05	0.55 ± 0.51	0.85 ± 0.11	0.47 ± 0.13
	Z- and *E*-Coutaric acid (µg/g)	201 ± 19	323 ± 43	232 ± 6	239 ± 15
	Z-Fertaric acid (µg/g)	40 ± 0.0b	97 ± 9c	22 ± 0.3a	23 ± 0.6a
	E-Fertaric acid (µg/g)	7.7 ± 0.1a	10 ± 2ab	14 ± 1b	18 ± 1c
	Syringic acid (µg/g)	0.13 ± 0.01	0.15 ± 0.01	0.20 ± 0.03	0.21 ± 0.03
FV	Aldehydes				
	Hexanal (ug/g)	33 ± 3ab	26 ± 5a	38 ± 1b	31 ± 2ab
	E-2-Hexenal (ug/g)	9.8 ± 0.8a	9.7 ± 1.8a	14 ± 0.4b	14 ± 1.9b
	1-Heptanal	22 ± 0.7b	18 ± 1a	25 ± 0.6b	22 ± 2b
	E-2-Octenal	9.1 ± 0.1b	5.7 ± 0.6a	9.6 ± 0.1b	9.5 ± 0.7b
	Nonanal	25 ± 3c	14 ± 1a	29 ± 0.7d	18 ± 0.4b
	E-2-Nonenal	19 ± 0.1c	15 ± 0.5a	18 ± 0.1c	17 ± 1b
	Benzaldehyde	2.9 ± 0.1ab	2.4 ± 0.3a	3.2 ± 0.4ab	3.5 ± 0.4b
	Furfural	0.33 ± 0.06b	0.31 ± 0.06b	0.07 ± 0.00a	0.10 ± 0.00a
	Isoprenoids				
	1,4-Cineole	0.054 ± 0.004a	0.054 ± 0.002a	0.049 ± 0.005a	0.085 ± 0.005b
	α-Terpinene	0.28 ± 0.03b	0.15 ± 0.00a	0.15 ± 0.06a	0.09 ± 0.01a
	γ-Terpinene	0.033 ± 0.004ab	0.038 ± 0.004b	0.024 ± 0.004a	0.025 ± 0.003a
	Terpinen-4-ol	0.29 ± 0.00b	0.073 ± 0.011a	0.065 ± 0.01a	0.091 ± 0.017a
	α-Terpineol	7.9 ± 0.02b	1.0 ± 0.2a	1.0 ± 0.3a	1.6 ± 0.5a
	β-Citronellol	0.052 ± 0.006c	0.041 ± 0.004bc	0.024 ± 0.002a	0.024 ± 0.004ab
	Linalool	7.3 ± 0.1c	7.1 ± 0.2c	5.9 ± 0.1a	6.4 ± 0.0b
	Nerol	0.096 ± 0.005bc	0.11 ± 0.01c	0.070 ± 0.001a	0.083 ± 0.008ab
	Geraniol	1.3 ± 0.1c	1.0 ± 0.13b	0.055 ± 0.06a	0.61 ± 0.11a
	C_{13}-apocarotenoids				
	β-Damascenone	0.024 ± 0.000c	0.023 ± 0.004bc	0.014 ± 0.000a	0.018 ± 0.000ab
	α-Ionone	0.016 ± 0.007	0.020 ± 0.010	0.010 ± 0.000	0.019 ± 0.007
	β-Ionone	0.44 ± 0.02c	0.32 ± 0.02b	0.26 ± 0.01a	0.25 0.03a
	Alcohols				
	1-Pentanol	51 ± 4ab	56 ± 0.5b	43 ± 6a	52 ± 4ab
	1-Hexanol	185 ± 16a	241 ± 36b	297 ± 3.4c	219 ± 11ab
	E-2-Hexenol	194 ± 2a	298 ± 28b	279 ± 6b	190 ± 23a
	Z-2-Hexenol	0.44 ± 0.00a	1.6 ± 0.1b	2.4 ± 0.2c	3.0 ± 0.3d
	E-3-Hexenol	2.1 ± 0.1a	4.1 ± 0.5b	11 ± 0.4d	6.7 ± 0.08c
	Z-3-Hexenol	21 ± 0.2a	44 ± 4b	48 ± 2b	45 ± 0.6b
	2-Ethyl-1-hexanol	8.5 ± 0.5	9.0 ± 0.6	8.4 ± 0.5	8.7 ± 0.0

1-Heptanol	0.40 ± 0.04ab	0.37 ± 0.08ab	0.28 ± 0.02a	0.42 ± 0.06b
1-Octanol	4.6 ± 1.3bc	6.4 ± 0.02c	2.7 ± 0.0a	3.7 ± 0.6ab
Benzyl alcohol	3.1 ± 0.1b	2.4 ± 0.5ab	1.7 ± 0.2a	3.4 ± 0.7b
2-Phenylethanol	7.5 ± 0.1a	7.5 ± 1.0a	5.6 ± 0.5a	11 ± 2b
Acetates				
Hexyl acetate	0.71 ± 0.05a	1.2 ± 0.2b	1.6 ± 0.2bc	1.7 ± 0.05c
Z-3-Hexenyl acetate	1.0. ± 0.0a	3.3 ± 0.0c	1.8 ± 0.1b	2.0 ± 0.2b
Others				
Hexanoic acid	133 ± 34	123 ± 10	129 ± 23	142 ± 51

[1]Different letters indicate significantly different groups according to Tukey's HSD comparison of means, *p*=0.05. [2]All compounds in ng/g unless indicated. Flavonoid-3-O-glc, Flavonoid-3-O-glucoside; Flavonoid-3-O-gal, Flavonoid-3-O-galactoside; Flavonoid-3-O-gln, Flavonoid-3-O-gluconoride.

Table S5. Data Blocks, ASCA Variable indices and K-means clustering

Variable Name	Data Block	ASCA Variable Index	K-Means cluster
Neoxanthin	CAR	1	2
Violaxanthin	CAR	2	1
Zeaxanthin	CAR	3	1
Lutein	CAR	4	2
β-Carotene	CAR	5	3
Chlorophyll *a*	CAR	6	2
Chlorophyll *b*	CAR	7	2
Pheophytin	CAR	8	2
Hexanal	FV	9	3
E-2-Hexenal	FV	10	3
1-Heptanal	FV	11	3
E-2-Octenal	FV	12	3
Nonanal	FV	13	3
E-2-Nonenal	FV	14	3
Benzaldehyde	FV	15	3
Furfural	FV	16	4
1,4-Cineole	FV	17	3
α-Terpinene	FV	18	4
γ-Terpinene	FV	19	4
Terpinen-4-ol	FV	20	4
α-Terpineol	FV	21	2
β-Citronellol	FV	22	4
Linalool	FV	23	4
Nerol	FV	24	4
Geraniol	FV	25	4
β-Damascenone	FV	26	4
α-Ionone	FV	27	-
β-Ionone	FV	28	4
1-Pentanol	FV	29	4
1-Hexanol	FV	30	3
E-2-Hexenol	FV	31	4
Z-2-Hexenol	FV	32	1
E-3-Hexenol	FV	33	1
Z-3-Hexenol	FV	34	1
2-Ethyl-1-hexanol	FV	35	-
1-Heptanol	FV	36	4
1-Octanol	FV	37	4
Benzyl alcohol	FV	38	4
2-Phenylethanol	FV	39	3
Hexyl acetate	FV	40	5
Z-3-Hexenyl acetate	FV	41	4
Hexanoic acid	FV	42	-

Quercetin 3-O-glc	PP	43	1
Quercetin 3-O-gal	PP	44	1
Quercetin 3-O-gln	PP	45	1
Laricitrin 3-O-glc	PP	46	4
Myricetin 3-O-glc	PP	47	1
Myricetin 3-O-gal	PP	48	4
Myricetin 3-O-gln	PP	49	5
Isorhamnetin 3-O-glc	PP	50	1
Isorhamnetin 3-O-gln	PP	51	1
Kaempferol 3-O-glc	PP	52	1
Kaempferol 3-O-gal	PP	53	1
Kaempferol 3-O-gln	PP	54	1
Z-Resveratrol	PP	55	5
E-Resveratrol	PP	56	4
Trimer	PP	57	4
Dihydroquercetin	PP	58	4
Astilbin	PP	59	1
Rutin	PP	60	1
Z-Piceid	PP	61	5
Naringenin chalcone	PP	62	5
(+)-Gallocatechin	PP	63	4
Procyanidin Dimer B1	PP	64	4
(-)-Epigallocatechin	PP	65	4
(+)-Catechin	PP	66	4
(-)-Epicatechin	PP	67	4
(-)-Epicatechin gallate	PP	68	1
Tyrosine	PP	69	-
Tryptophan	PP	70	-
Phenylalanine	PP	71	4
Glutathione reduced	PP	72	4
Glutathione oxidised	PP	73	1
Z-Caftaric acid	PP	74	4
E-Caftaric acid	PP	75	4
Z- and *E*-Coutaric acid	PP	76	4
p-Coumaric acid	PP	77	-
Z-Fertaric acid	PP	78	4
E-Fertaric acid	PP	79	5
Syringic acid	PP	80	-
